# Molecular diagnostic and genetic characterization of highly pathogenic viruses: application during Crimean–Congo haemorrhagic fever virus outbreaks in Eastern Europe and the Middle East

**DOI:** 10.1111/1469-0691.12075

**Published:** 2012-12-14

**Authors:** C Filippone, P Marianneau, S Murri, N Mollard, T Avsic-Zupanc, S Chinikar, P Desprès, V Caro, A Gessain, N Berthet, N Tordo

**Affiliations:** 1 Institut Pasteur, Unit of Epidemiology and Pathophysiology of Oncogenic VirusesParis, France; 2 Department of Virology, Institut Pasteur, URA CNRS URA 3015Paris, France; 3 Virology Unit, Laboratoire de Lyon, ANSESLyon, France; 4 Institut Pasteur, Unit of Biology of Viral Emerging Infection, National-WHO-OIE Reference Centre for Viral Haemorrhagic FeversLyon, France; 5 Medical Faculty, Institute of Microbiology and ImmunologyLjubljana, Slovenia; 6 Institut Pasteur of Iran, Arboviruses and Viral Haemorrhagic Fevers Laboratory, National Reference LaboratoryTeheran, Iran; 7 Institut Pasteur, Unit of Molecular Interactions Flavivirus–Host, WHO-OIE Reference Centre for Viral Haemorrhagic FeversParis, France; 8 Institut Pasteur, Genotyping of Pathogens and Public HealthParis, France; 9 Institut Pasteur, Antiviral Strategies Unit, WHO-OIE Reference Centre for Viral Haemorrhagic FeversParis, France

**Keywords:** Crimean–Congo haemorrhagic fever virus, differential diagnosis, microarray, viral haemorrhagic fevers, viral zoonoses

## Abstract

Several haemorrhagic fevers are caused by highly pathogenic viruses that must be handled in Biosafety level 4 (BSL–4) containment. These zoonotic infections have an important impact on public health and the development of a rapid and differential diagnosis in case of outbreak in risk areas represents a critical priority. We have demonstrated the potential of a DNA resequencing microarray (PathogenID v2.0) for this purpose. The microarray was first validated *in vitro* using supernatants of cells infected with prototype strains from five different families of BSL-4 viruses (e.g. families *Arenaviridae, Bunyaviridae, Filoviridae, Flaviviridae and Paramyxoviridae*). RNA was amplified based on isothermal amplification by Phi29 polymerase before hybridization. We were able to detect and characterize Nipah virus and Crimean–Congo haemorrhagic fever virus (CCHFV) in the brains of experimentally infected animals. CCHFV was finally used as a paradigm for epidemics because of recent outbreaks in Turkey, Kosovo and Iran. Viral variants present in human sera were characterized by BLASTN analysis. Sensitivity was estimated to be 10^5^–10^6^ PFU/mL of hybridized cDNA. Detection specificity was limited to viral sequences having ∼13–14% of global divergence with the tiled sequence, or stretches of ∼20 identical nucleotides. These results highlight the benefits of using the PathogenID v2.0 resequencing microarray to characterize geographical variants in the follow-up of haemorrhagic fever epidemics; to manage patients and protect communities; and in cases of bioterrorism.

## Introduction

Viruses recognized as highly pathogenic for humans must be manipulated in a Biosafety level 4 (BSL-4) laboratory. They include viruses associated with encephalitis and respiratory infections, such as recently emerged members of the genus *Henipavirus*, family *Paramyxoviridae* and haemorrhagic fever viruses in the families *Arenaviridae, Filoviridae, Bunyaviridae* and *Flaviviridae* 1. Infections with these viruses lead to a wide spectrum of clinical outcomes, from flu-like and malaria-like symptoms to vascular complications that may cause death 1,2. Most members of the genus *Flavivirus* (family *Flaviviridae*) are arthropod-borne, as are those of the family *Bunyaviridae*, except for the genus *Hantavirus* which is rodent-borne or insectivore-borne 2,3. Viruses of the family *Arenaviridae* are also rodent-borne 2. Those of the genus *Henipavirus* have bat reservoirs but may also infect humans through contact with infected horses or pigs 4. Recent data indicate that bats are also probable reservoirs and vectors for viruses of the family *Filoviridae* 5,6. Interhuman transmission and nosocomial infections also contribute to spreading the diseases 2,7.

Development of vaccines to prevent infection by these emerging zoonotic viruses is limited and only ribavirin has been used as an efficacious treatment for several of them 1, so early, rapid and specific diagnosis is critically important for disease control. At-risk areas should possess the necessary facilities and equipment, as well as rapid tests, to be prepared for public health emergencies 2,8. Accurate diagnoses have traditionally relied on specific serological and virological analyses, which include western blotting, ELISA, immunofluorescence staining, genome detection by PCR and quantitative PCR, and ultimately, virus isolation 9–13. Molecular methods are rapid and specific but are limited by the high genetic variability among different viral strains. To overcome this limitation, macroarray and microarray technology platforms have been developed to detect and identify a large number of pathogens in a single assay 14–20. Long oligonucleotide probes have been used previously for the detection of viruses associated with haemorrhagic fevers 16. Low-density macroarrays allowed different variants of Crimean–Congo haemorrhagic fever virus (CCHFV) to be rapidly detected 17, but were complicated by a requisite reverse transcription (RT-) PCR step. High-density resequencing microarrays not only detect pathogens but also determine nucleic acid sequences to single base-pair resolution. A large panel of viral genome sequences from different geographical origins can be characterized in a single test. The high-density resequencing DNA microarray, PathogenID v2.0, has been shown to be useful for rapid diagnosis during emerging viral infections, such as the 2009 influenza pandemic 18, and was useful for genotyping members of the family *Rhabdoviridae* 19.

Here, we used the PathogenID v2.0 microarray to detect highly pathogenic viruses. We first validated the microarray with *in vitro* samples by analysing supernatants from cells infected by prototype virus strains and variants belonging to five families of BSL-4 agents (*Arenaviridae, Bunyaviridae, Filoviridae, Flaviviridae, Paramyxoviridae*). We then evaluated its performance during a health emergency situation by testing human sera from CCHFV outbreaks in Turkey (2009), Kosovo (2001) and Iran (2009). CCHFV belongs to the genus *Nairovirus*, family *Bunyaviridae* and has the largest geographic distribution among haemorrhagic fever viruses 21,22. Zoonotic infection occurs either directly through its vectors, which are various tick species from the genus *Hyalomma*, or indirectly through contact with infected livestock. Hospital environments are also vulnerable to inter-human transmissions 23. CCHFV infection is associated with several clinical outcomes, some of which can become life threatening 22. CCHFV outbreaks or sporadic cases have occurred in Mauritania 24, Iran 10, Turkey 25, Kosovo 26 and Sudan 23.

## Materials and Methods

### Ethics statement

This work includes a retrospective study on 12 human sera from clinical specimens submitted to France National-WHO-OIE Reference Centres for diagnosis during CCHF epidemics in Kosovo, Turkey and Iran.

The collection of the remaining samples to be used for scientific purpose was declared to and approved by the Comité de Protection des Personnes, Ile-de-France I and the French Research Ministry (no. DC 2011-1471) according to French regulations.

Animal experimental methods were approved by the Région Rhône Alpes Ethics Committee (France).

### Viruses

Viral strains and geographical variants (Table 1 and Table 2) were cultured and isolated in permissive Vero-E6 cells as previously described 11,27. To simulate the complexity of clinical samples, we pooled RNA samples from different Vero-E6 cell cultures that had each been infected by a single virus. Twelve pooled RNA samples of one to three viruses each were prepared. For Junin virus (family *Arenaviridae*) and Sin Nombre virus (genus *Hantavirus*, family *Bunyaviridae*) synthetic cDNA sequences (Eurofins MWG Operon, Ebersberg, Germany) were used as templates for the amplification step.

**TABLE 1:** Sequences of the RNA-dependent RNA polymerase genes of highly pathogenic viruses tiled on the PathogenID v2.0 microarray

Family	Genus	Species	Subtype/Strain	Tiled sequence size (nucleotides): location along the L segment (accession no.)
*ARENAVIRIDAE*	*Arenavirus* (Old World)	*Lassa virus*	Josiah—Sierra Leone	525: 4259–4783 (U63094.1)
	
	*Arenavirus* (New World)	*Machupo virus*	Carvallo—Bolivia	528: 2469–2996 (AY358021.2)
		
		*Guanarito virus*	INH-95551—Venezuela	528: 4099–4626 (AY216504.2)
		
		*Junin virus*	XJ13—Argentina	528: 2462–2989 (FJ805377.1)

*BUNYAVIRIDAE*	*Nairovirus*	*Crimean–Congo haemorrhagic fever virus*	IbAr10200—Nigeria	531: 2717–3247 (AY389361.2)
	
	*Hantavirus*	*Hantaan virus*	76–118—Korea	510: 3131–3640 (X55901.1)
		
		*Puumala virus*	Sotkamo—Finland	531: 4705–5235 (Z66548.1)
		
		*Seoul virus*	80–39—South Korea	552: 3055–3606 (X56492.1)
		
		*Dobrava-Belgrade virus*	DOBV/Ano-Poroia/Af19/1999—Greece	531: 3905–4435 (AJ410617.1)
		
		*Sin Nombre virus*	NM R11—New Mexico	528: 4857–5384 (L37902.1)
	
	*Phlebovirus*	*Rift Valley fever virus*	MP-12—Egypt Sharqiya	549: 5026–5574 (DQ375404.1)

*FLAVIVIRIDAE*	*Flavivirus*	*Kyasanur Forest disease virus*	KFD P 9605—India	504: 8463–8966 (HM055369.1)
		
		*Yellow fever virus*	17D RKI—vaccine strain	504: 8429–8932 (JN628279.1)
		
		*Tick-borne encephalitis virus*	Neudoerfl—Austria	501: 134–634^a^ (EU303230.1)

*FILOVIRIDAE*	*Ebolavirus*	*Reston ebolavirus*	Pennsylvania	528: 13611–14138^b^ (AF522874.1)
		
		*Zaire ebolavirus*	Mayinga—1976	528: 13642–14169^b^ (AF086833.2)
	
	*Marburgvirus*	*Marburg marburgvirus*	Popp	528: 2266–2793 (X68494.1)

*PARAMYXOVIRIDAE*	*Henipavirus*	*Nipah virus*	UMMC1—Malaysia	528: 13743–14270^b^ (AY029767.1)
		
		*Hendra virus*	Australia	528: 13731–1425^b^ (AF017149.2)

a Location referred to the NS5 gene.

b Location referred to the entire genome.

**TABLE 2:** Microarray detection of prototype viruses and geographical variants

				Detection in mixture^b^											
				
				1	2	3	4	5	6	7	8	9	10	11	12
				
Sequence tiled	Viral strain/Variant tested	Identity tiled seq/virus seq (%)	Call rate^a^(%)												
Lassa virus Josiah—Sierra Leone	Josiah—Sierra Leone^c^	100	98.4				X								
	
	Ivory Coast (AV)^c^	81	28.7					X							
	
	Guinea^c^	NA	98.8						X						

Junin virus XJ13—Argentina	XJ13^d^	100	98.0												X

Crimean–Congo haemorrhagic fever virus IbAr10200—Nigeria	IbAr10200, Nigeria^c^	100	99.6		X										
	
	Ar-39554, Mauritania^c^	98	98.0				X								
	
	Tokat 2003, Turkey^c^	89	62.9	X											
	
	BA66019, China^c^	86	30.9			X									

Hantaan virus 76–118—Korea	76–118 Korea^c^	100	97.3							X					

Sin Nombre virus NM R11—New Mexico	NM R11—New Mexico^d^	100	99.8											X	

Seoul virus 80–39—South Korea	Tchoupitoulas virus^c^	98	98.3						X						

Dobrava-Belgrade virus															

DOBV/Ano-Poroia/Af19/1999—Greece	Slovenia 3970/87^c^	94	65.9									X			

Rift Valley fever virus MP-12															

Egypt—Sharqiya	ZH548—Egypt^c^	99	97.3						X						

Kyasanur Forest disease virus															

KFD P 9605—India	Alkhurma virus^c^	92	73.9	X											

Tick-borne encephalitis virus															

Neudoerfl—Austria	Omsk haemorrhagic fever virus Balangul^d^	82	41.7			X				X					

Reston ebolavirus—Pennsylvania	Reston^c^	100	98.6		X										

Zaire ebolavirus Mayinga—1976	Zaire, 1995^c^	100	94.8							X					
	
	Gabon, 2001^c^	99	91.3								X				

Marburg marburgvirus Popp	Popp, Uganda, 1967^c^	100	98.4				X								
	
	Musoke-Kenya,1880^c^	95	98.2					X							

Nipah virus UMMC1—Malaysia	Malaysia^c^	100	99.6		X							X			

Hendra virus—Australia	Australia^c^	100	99.4	X									X		

a Call rate for the detection of the strain/isolate on the microarray.

b Mixtures of RNA extracted from different cell cultures infected with different viruses. For each mix, the viral RNA present is identified by a X.

c Detection in infected cell supernatants.

d Detection of synthetic sequence.

NA, sequence not available.

### Human sera from CCHFV outbreaks

Sera from 12 infected humans were collected during CCHFV outbreaks (2003–09) in the Balkans (five from Kosovo, 2001 and two from Turkey, 2009) and the Middle East (five from Iran, 2009).

### Animal biopsies

One non-human primate, a New World squirrel monkey (*Saimiri sciureus*) was experimentally infected intravenously with 10^3^ PFU UM-MC1 Malaysian isolate of Nipah virus 28 as previously described 29. It was imported from a breeding colony in French Guiana and housed in the BSL-4 animalcare facility in Lyon. The animal was observed daily for signs of disease onset; disease symptoms appeared at day 10 and lasted for 3 days before the moribund monkey was humanely euthanized. A brain biopsy was taken at necropsy and frozen at −80°C.

In another experiment, ten newborn Swiss mice were intracranially inoculated with 20 µL CCHFV (i.e. 10^3^ PFU) each in the BSL-4 animal-care facility in Lyon. Seven days after infection, mice were euthanized. Brain was collected, crushed in phosphate-buffered saline 1 × (1/10 weight/volume), and clarified by centrifugation for 15 min at 600 ***g*** before storage at −80°C.

### RNA extraction

RNA extraction was performed using the QIAamp Viral RNA Mini Kit (Qiagen Inc., Valencia, CA, USA) as previously described 11. For BSL-4 viruses, the cell lysis step was carried out at the Jean Mérieux BSL-4 Laboratory (Lyon, France) according to the validated BSL-4 procedure.

### Amplification of viral RNA

Extracted viral RNAs were reverse transcribed into cDNA using SuperScript III reverse transcriptase (Invitrogen Inc., Carlsbad, CA, USA) then amplified by the whole transcriptome amplification (WTA) approach in the presence of random hexamer primers. An optimized protocol based on isothermal amplification by the Phi29 polymerase was applied to the QuantiTect Whole Transcriptome Kit (Qiagen) as previously described 30.

### Quantitative RT-PCR and PCR

Quantitative RT-PCR and PCR amplifications of CCHFV sequences present in infected cell supernatants or human sera were performed in a Light-Cycler Instrument (Roche Applied Sciences, Basel, Switzerland) 31. Treated samples were: (i) extracted RNA, (ii) cDNA obtained following reverse transcription of extracted RNA using random primers, and (iii) WTA products obtained following amplification by Phi29 polymerase.

### Hybridization to PathogenID v2.0 microarray and data analysis

The PathogenID v2.0 microarray is the second generation of a microarray developed through a collaboration between Affymetrix and Institut Pasteur 19,30. It was designed to detect 949 genes, including 126 different viral sequences 18,19, 18 of which correspond to highly pathogenic viral agents (Table 1).

**FIG. 1:**
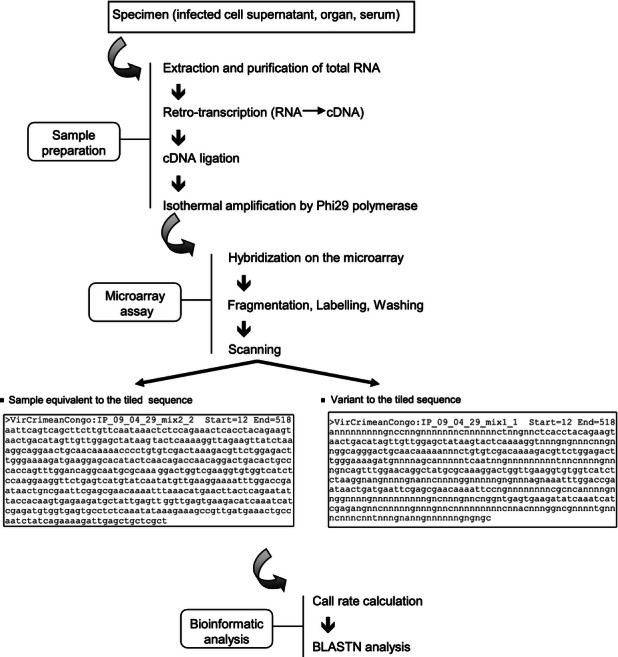
Flow chart of the experimental procedure based on resequencing microarrray for the detection of highly pathogenic viruses.

The entire microarray experimental procedure is summarized in Figure 1. Total cDNA (20–25 µg in 25 µL) that had been amplified from 100 µL of cell culture supernatant or from 25 µL of a serum sample was fragmented, labelled and hybridized overnight at 45°C to the PathogenID v2.0 microarray. The array was then washed and scanned according to instructions provided by Affymetrix. Results were analysed using GeneChip Operating Software version 4.0 (GCOS), GeneChip Sequence Analysis Software version 4.0 (GSEQ), and the ABACUS algorithm 32.

The call rate value (the percentage of nucleotides identified by the microarray) obtained from each sample hybridized on the microarray was used to determine the degree of hybridization of that sample and to compare it with that of other samples. All the obtained sequences were exported into a FASTA-formatted file and then subjected to BLASTN analysis to identify viral variants.

After scanning and analysis, all the chips were destroyed according to BSL-4 waste guidelines.

### Direct sequencing

All specimens used either for the validation steps of the PathogenID v2.0 microarray or for clinical investigation of the outbreaks, were sequenced directly. To analyse the CCHFV strains, classical, nested or semi-nested PCR were performed to amplify the region tiled on the microarray, e.g. the 531 bases of the L segment encoding the RNA-dependent RNA polymerase. Degenerate primer design and sequence analysis were performed using MacVector software (MacVector Inc., Cary, NC, USA). Primer position refers to the L genome segment of the prototype CCHFV strain (IbAr10200): fw2645 (5′-TGCTCWTTYATTGCCTGTGC-3′); rev3269 (5′-TNACACCRTTGGGGTGACA-3′); fw2576(5′-GGGAAAATAAGGACAGACCA-3′); rev3371 (5′-TCYGTTAAGCATTCATTRCT-3′). The PCR fragments were purified by ultrafiltration before sequencing (Millipore, Billerica, MA, USA). Sequencing was performed using a BigDye Terminator v1.1 cycle sequencing kit (Applied Biosystems, Carlsbad, CA, USA) and purified by ethanol precipitation. Sequence chromatograms from both strands were obtained on an automated sequence analyser ABI3730XL (Applied Biosystems) with the PCR primers. The percentage of sequence divergence was calculated for each sample by determining the number of mutations relative to the prototype sequence tiled on the microarray.

### Phylogenetic analysis

A phylogenetic analysis of CCHFV sequences was performed by the neighbour-joining method using BioNumerics software for Windows (version 5.1, Applied-Maths, Sint-Martens-Latem, Belgium). The sequences used for this purpose were: (i) all the RNA-dependent RNA polymerase L sequences available in GenBank, (ii) the sequences obtained by direct sequencing and (iii) the sequences obtained from the microarray results.

## Results

We used the high-density PathogenID v2.0 resequencing microarray to detect and identify a number of different highly pathogenic viruses. This work was divided into two parts: (i) a validation step, in which we used supernatants from cultured cells infected with viral strains that matched the prototype probes tiled on the microarray and their variants, and (ii) an exploratory step, in which we used human sera from CCHFV outbreaks to evaluate the potential of the microarray to be used in public health emergencies.

### Detection and differential diagnosis of viral prototype strains and their variants

We assessed whether the PathogenID v2.0 microarray could be used to correctly detect and identify different viruses present in a single sample designed to resemble a complex biological specimen that might occur in nature or the laboratory (i.e. screening a pool of samples). Hence, total RNA was extracted from the supernatants of cells that had been infected with a single viral strain. Then, pools of RNA from up to three different supernatants were made to resemble likely combinations that might coexist in the same geographical area or animal host (Table 2). For two viruses that were absent from our laboratory collection (Junin virus and Sin Nombre virus), two plasmids encompassing the synthetic sequences tiled on the microarray were introduced into certain pools after the reverse transcription step and were then further amplified by WTA.

The microarray detected and characterized each virus prototype to similar levels of sensitivity whether the viral RNA was tested alone or in a pool (Table 2). Similar results were obtained when viruses were mixed before RNA extraction 18. These results indicate that detection of one virus was not affected by the presence of one or two others. For the family *Arenaviridae*, the Junin virus plasmid clearly validated the homologous sequence on the microarray (call rate: 98%). For Old World viruses of the family *Arenaviridae*, the tiled Lassa virus sequence (Josiah, Sierra Leone) detected the homologous strain (call rate: 98.4%) and a variant from Guinea (call rate: 98.8%). In addition, even a divergent variant from Ivory Coast (AV) was significantly detected (call rate: 28.7%). Among the family *Bunyaviridae*, the Sin Nombre virus NM–R11, Hantaan virus 76-118, Rift Valley fever virus ZH548 and CCHFV IbAr10200 hybridized to their homologous sequences as expected (call rates: 99.8%, 97.3%, 97.3% and 99.6%, respectively). Moreover, the tiled Nigerian CCHFV IbAr10200 sequence also detected CCHFV variants from Mauritania, Turkey and China (call rates: 98%, 62.9% and 30.9%, respectively). Similarly, the tiled Dobrava-Belgrade virus sequence (DOBV/Ano-Poroia/Af19/1999) detected the variant 3970/87 from Slovenia (call rate: 65.9%) whereas the Seoul virus sequence detected the related Tchoupitoulas virus (call rate: 98.3%). For the family *Filoviridae*, the tiled Popp strain of Marburg marburgvirus was as efficient for detection of the Musoke strain (call rate: 98.2%) as for the homologous strain (call rate: 98.4%). The tiled Reston ebolavirus and Zaire ebolavirus sequences allowed detection of the homologous species (call rate: 98.6% and 94.8%, respectively) and an additional variant from Gabon 2001 (call rate: 91.3%). Among the family *Flaviviridae*, the tiled sequences from the Kyasanur Forest disease virus KFD P 9605 and tick-borne encephalitis virus Neudoerfl detected the heterologous Alkhurma virus (call rate: 73.9%) and Omsk haemorrhagic fever virus Balangul (call rate: 41.7%), respectively. Finally for the genus *Henipavirus* (family *Paramyxoviridae*) Nipah virus Malaysia and Hendra virus Australia were perfectly detected by the homologous sequence (call rates: 99.6% and 99.4%, respectively).

In summary, the PathogenID v2.0 resequencing microarray very efficiently detected: (i) prototype virus strains and the two synthetic probes with excellent call rates (>97%); (ii) variants with high call rates similar to those of prototype strains (e.g. Lassa virus Guinea, CCHFV Mauritania, Tchoupitoulas virus, Marburg marburgvirus Musoke, Zaire ebolavirus Gabon); (iii) variants with moderate call rates (e.g. 41.6%, for Omsk haemorrhagic fever virus). Variants with low call rates (30.9% for CCHFV from China, 28.7% for Lassa virus from Ivory Coast) were also detected, although less significantly. Finally, highly divergent variants were not detected by the microarray (data not shown).

### Application of the microarray to CCHFV outbreaks

**FIG. 2:**
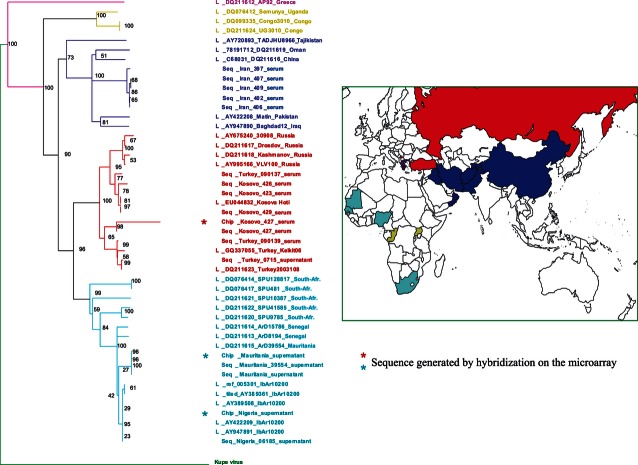
Phylogenetic Tree. Phylogenetic analysis of Crimean–Congo haemorrhagic fever virus (CCHFV) variants was performed using a 531-bp sequence in the CCHFV L segment encoding RNA-dependent RNA polymerase (position 2717–3248) and the neighbour-joining method with BioNumerics software for Windows (version 5.1, Applied Maths). Sequences were: (i) retrieved from GenBank (L); (ii) experimentally obtained from supernatants of CCHFV-infected cell cultures or from infected human serum (Seq); or (iii) the results output from the microarray (Chip) (*).

We next evaluated the ability of the microarray to detect viruses in human serum samples that were collected during virus outbreaks. CCHFV was chosen as an example because this virus has emerged several times in recent years, particularly in Eastern Europe (Balkan region) and the Middle East. We used (i) sera from 12 CCHFV-positive patients from recent outbreaks in Turkey (2009), Kosovo (2001) and Iran (2009); and (ii) four CCHFV strains (Nigeria, Mauritania, Turkey and China) grown in cell culture (Table 2). We sequenced 531 bp of the polymerase gene of each strain/isolate and constructed a phylogenetic tree that also included all the CCHFV sequences available in GenBank (Fig. 2). Phylogenetic analysis distinguished five genetic clusters, as has been previously described 26,33,34. Two clusters are in Africa: one is spread from western (Mauritania, Senegal and Nigeria) to southern Africa (South African Republic) and includes the Nigerian and Mauritanian sequences; the other is restricted to Equatorial Africa (Congo, Uganda). A Eurasian cluster spreads from Kosovo/Turkey northward to Russia. A Middle East cluster comprises samples from Oman, Iraq, Pakistan, Tajikstan to China. Concerning the viruses present in the human sera we tested, the Iranian viruses formed a distinct branch in the Middle East cluster whereas those from Kosovo and Turkey segregated in two sub-branches of the Eurasian cluster: one together with the Kosovo Hoti strain (Kosovo 423, 426, 429 and Turkey 090137); the other with the Turkey Kelkit06 and 200310849 strains (Turkey 090139 and Kosovo 427).

The microarray clearly detected three out of the four CCHFV reference strains, the China strain being only poorly detected (call rate 30.9% but no BLASTN confirmation). It also allowed the geographical characterization of five out of the 12 CCHFV serum samples: two samples from Turkey and three from Kosovo, all belonging to the Eurasian cluster (Table 3). The two remaining Kosovo samples and all five samples from Iran were not detected.

**TABLE 3:** Quantitative evaluation of the different steps of the microarray procedure for the detection of clinical serum samples from Crimean–Congo haemorrhagic fever virus (CCHFV) outbreaks

Sample	RNA (PFU/mL)^a^	cDNA (PFU/mL)^a^	WTA (PFU/mL)^a^	Call rate^b^ (%)	BLASTN (Homologous Strain)	Divergence^c^ versus homologous strain (%)
Nigeria (supernatant)	5.9 × 10^5^	8.5 × 10^4^	1.0 × 10^10^	99.6	IbAr10200	0.2
Mauritania (supernatant)	8.1 × 10^6^	1.8 × 10^5^	2.8 × 10^12^	98.0	ArD39554	1.9
Turkey (supernatant)	6.2 × 10^6^	1.5 × 10^5^	1.4 × 10^11^	62.9	Turkey200310849/Kelkit06	10.7
China (supernatant)	9.1 × 10^5^	2.2 × 10^6^	5.0 × 10^8^	30.9	–	13.7
Turkey 090137 (serum)	1.2 × 10^3^	2.6 × 10^3^	5.6 × 10^7^	45.7	Eurasia	10.15
Turkey 090139 (serum)	7.6 × 10^1^	2.4 × 10^1^	9.9 × 10^5^	33.5	–	10
Kosovo 422 (serum)	1.2 × 10^1^	2.4 × 10^0^	6.1 × 10^5^	39.0	Eurasia	ND
Kosovo 423 (serum)	2.3 × 10^0^	1.9 × 10^0^	3.5 × 10^4^	22.9	–	9.2
Kosovo 426 (serum)	1.1 × 10^2^	1.4 × 10^1^	8.1 × 10^4^	ND	ND	9.8
Kosovo 427 (serum)	1.8 × 10^3^	1.3 × 10^3^	1.9 × 10^6^	70.8	Turkey200310849/Kelkit06	9.8
Kosovo 429 (serum)	2.6 × 10^3^	7.0 × 10^2^	2.8 × 10^5^	29.2	Eurasia	9.7
Iran 397 (serum)	7.0 × 10^2^	8.5 × 10^2^	2.5 × 10^7^	23.5	–	14.1
Iran 402 (serum)	3.2 × 10^4^	1.8 × 10^3^	3.9 × 10^8^	26.2	–	14.7
Iran 406 (serum)	1.8 × 10^4^	2.9 × 10^3^	3.6 × 10^8^	24.0	–	14.5
Iran 407 (serum)	4.0 × 10^4^	3.2 × 10^3^	5.0 × 10^8^	28.4	–	14.7
Iran 409 (serum)	4.7 × 10^3^	1.9 × 10^3^	8.9 × 10^6^	21.5	–	14.7

a Specific viral genetic material evaluated by quantitative PCR, expressed in equivalent PFU/mL.

b Call rate for the detection of the strain/isolate on the microarray.

c Percentage of divergence (531 base pairs region in the polymerase gene) against the sequence tiled on the microarray.

No BLASTN confirmation. ND not done.

To determine why these samples were not detected, we characterized the viral genetic material at each step of the detection process (Table 3). We used quantitative PCR to precisely measure the amounts of specific viral genetic material present before and after RNA amplification. The amount of viral RNA in each original sample was comparable to or slightly higher than (±l0^1^ maximum) the amount of specific cDNA after random priming. This indicated that reverse transcription did not substantially affect the amount of specific viral genetic material. In contrast, the WTA isothermal amplification of cDNA by Phi29 polymerase significantly increased this amount. The increase from the original amount of RNA in the sample to the cDNA after WTA was between 1.08 × 10^2^ PFU/mL to 3.46 × 10^5^ PFU/mL, with a mean increase of 3.72 × 10^4^ PFU/mL. Interestingly, the lower amount of amplified cDNA detected by the microarray was 2.8 × 10^5^ PFU/mL for the Kosovo sample 429 (Kosovo samples 423 and 426 with 3.5 × 10^4^ PFU/mL and 8.1 × 10^4^ PFU/mL respectively, were not detected). On the other hand, the Turkey sample 090139 with 9.9 × 10^5^ PFU/mL was not detected. As Kosovo and Turkey samples are equally divergent from the tiled sequence (∼10%; Table 3), the sensitivity detection limit of the microarray must therefore be estimated between 10^5^ and 10^6^ PFU/mL of amplified cDNA.

Limited genetic material did not explain why the five samples from Iran were not detected, because the amounts of amplified cDNA hybridized on the microarray (8.9 × 10^6^ to 5.0 × 10^8^ PFU/mL) were all well above the 10^5^/10^6^ PFU/mL detection limit (Table 3). The China strain was poorly detected despite the presence of sufficient material hybridized (5.0 × 10^8^ PFU/mL). Therefore, a degree of divergence of about 13.7–14.7% from the tiled sequence (Nigerian IbAr10200 strain) is the specificity detection limit of the microarray.

For the sequences detected by the microarray, call rate values were between 29.2% (Kosovo 429) and 70.8% (Kosovo 427), which were globally lower than those obtained from the infected cell supernatants (62.9–99.6%). Nevertheless, the BLASTN analysis allowed the geographical origin of the different isolates to be assessed with a precision dependent on the quality of the call rate. Sequences from samples having call rates >70% were precisely segregated into their specific sub-cluster in the phylogenetic tree along with sequences obtained by their direct sequencing (Fig. 2). This is the case for the Nigeria and Mauritania strains and for the Kosovo 427 serum (Eurasian sub-cluster). The only difference consisted of a longer branch on the tree that was proportional to the number of nucleotides undetermined by the microarray. For the Kosovo 429 and Turkey 090137 sera, which yielded lower call rates (29.2% and 45.7%, respectively), the analysis nevertheless specified that they belonged in the Eurasian cluster.

### Application of the microarray to infected animal brain

The capacity of the microarray to detect viruses in animal samples was tested (Table 4). CCHFV was detected in the brain of newborn mice experimentally infected intracranially for the purpose of virus isolation. The amplification of the cDNA by Phi29 was even more efficient than for human serum samples (increase ratio of 10^5^ from the original RNA, 10^4^ from the cDNA), which indicates that the complexity of genetic material of the sample did not impair WTA amplification. However, the call rate was lower than for supernatants of cells infected with the same CCHFV IbAr10200 strain (82.6% versus 99.6%), suggesting a higher background for hybridization (Table 3).

**TABLE 4:** Quantitative evaluation of the different steps of the microarray procedure for viral detection in brain samples from experimentally infected animals

Sample	RNA (PFU/mL)^a^	cDNA (PFU/mL)^a^	WTA (PFU/mL)^a^	Call rate^b^ (%)	BLASTN (Homologous Strain)	Divergence^c^ versus homologous strain (%)
CCHFV IbAr10200 (mouse brain)	1.9 × 10^4^	2.0 × 10^5^	1.9 × 10^9^	82.6	IbAr10200	1.9
Nipah virus UMMC1 (monkey brain)	3.08 × 10^5^	4.4 × 10^5^	2.3 × 10^9^	60.9	UM-MC1	ND

CCHFV, Crimean–Congo haemorrhagic fever virus; ND, not done.

a Specific viral genetic material evaluated by quantitative PCR, expressed in equivalent PFU/mL.

b Call rate for the detection of the strain/isolate on the microarray.

c Percentage of divergence (531 bp region in the polymerase gene) against the sequence tiled on the microarray.

In addition, the neurotropic Nipah virus was detected in the brain of a monkey moribund upon an experimental intravenous infection. As observed above, the complexity of genetic material in the sample did not significantly affect the amplification of viral material (increase ratio of 5.2 × 10^3^ from the cDNA) but generated a higher background for hybridization (call rate 60.9% versus 99.6%) compared with that obtained with cell supernatant infected with the same viral strain (Table 3).

## Discussion

Highly pathogenic viruses are endemic in developing countries where their impact on public health is especially important in light of the absence of efficacious treatments and vaccines 1. Occasionally, they can be brought into the developed world by travellers and could be misused for bioterrorism. These viruses produce haemorrhagic fevers, encephalitis or respiratory symptoms, but their aetiology is hard to establish in the absence of specific clinical symptoms. Hence, rapid differential diagnosis during outbreaks represents a critical public health priority.

Among the molecular techniques used in clinical and field diagnosis, (RT)-PCR is considered a reference standard because of its versatility and rapid turnover. However, it may also be limited by pitfalls such as the genetic variability of the viral isolates or doubtful aetiology requiring the design of a battery of specific or degenerated primers, etc. Under these conditions, DNA microarray technology offers the advantage of performing a differential diagnosis in a single test. It has already proven effective for pathogen detection and epidemiological studies 14,20,35. The GreeneChip 60-mer oligonucleotide array provided a good level of sensitivity for the diagnosis of different infections including viral haemorrhagic fevers, but was problematic because it required correction of probe intensities and subtraction of the negative control 16. The resequencing microarray approach rapidly identifies virus variants while simultaneously characterizing their genome sequences 20. The confidence levels of these data depend on the virus's similarity to a tiled reference sequence 36. It is a promising diagnostic alternative for RNA viruses which have high levels of genetic variability 15,19,37. The PathogenID v2.0 resequencing microarray has precisely identified the geographic origin of virus isolates, which is crucial for monitoring an epidemic or a pandemic 18. It has also been used to help in genotyping of viruses for taxonomic purposes 19. In our study, we evaluated the ability of this microarray to detect variants of highly pathogenic BSL-4 viruses from the families *Arenaviridae, Bunyaviridae, Flaviviridae, Filoviridae* and *Paramyxoviridae*. We first validated its spectrum in differential diagnosis, then explored its potential in sensitivity and specificity for use with human serum samples from CCHFV outbreaks in Eastern Europe (Balkan region) and the Middle East.

Validation was performed using different types of samples (i.e. cell supernatants, human sera and animal brain) at different degrees of complexity and divergence from the tiled sequence. In single analyses containing multiple virus types, the microarray was able to identify specific viruses among pathogens that produce similar symptoms, and to discriminate between variants of different origins. This is crucial for clinical management of outbreaks that may involve viruses, bacteria or parasites 1,2,9. The ∼48-h procedure required to complete the assay may appear less rapid than classical PCR-based methods. However, when a differential diagnosis is needed for an unknown aetiology, the PathogenID v2.0 microarray might be competitive because it does not require (i) designing specific primers for all potential etiologic agents, (ii) setting up the corresponding PCR assays, and (iii) performing the sequence and bioinformatic analyses.

Crimean–Congo haemorrhagic fever virus was chosen as a model infectious agent with which to test the microarray because it has a widespread geographic distribution 8,10,21,22 and substantial genetic diversity 24,25,33,34,38–40. The Nigerian strain (from the African cluster 41 tiled on the microarray: (i) perfectly detected variants of the same African cluster (e.g. Mauritania, call rate: >97%); (ii) correctly identified viruses of the Eurasian cluster, which are about 9% divergent (e.g. Turkey, call rate: 70%); and (iii) weakly detected viruses from the Middle East cluster (China, call rate: 30.9%). Analysis of human sera from recent epidemics in Kosovo, Turkey and Iran clearly demonstrated the utility of this microarray for detection and characterization by phylogenetic analysis of viruses circulating during outbreaks (Table 3 and Fig. 2). For example, it showed that variants from two sub-groups of the Eurasian cluster were co-circulating in the Balkan region (Kosovo/Turkey), which confirmed previous observations 42.

Using quantitative RT-PCR 28, the microarray sensitivity limit was estimated to be between 10^5^ and 10^6^ PFU/mL of hybridized cDNA per sample. As the mean amplification ratio from the original RNA to the cDNA after WTA was 3.72 × 10^4^ (considering all samples) and 1.75 × 10^4^ (only considering serum samples), the detection limit of the microarray is between 10^1^ and 10^2^ equivalent PFU of original viral RNA per mL of serum. This compares favourably with the sensitivity limit of the quantitative RT-PCR method described by Wölfel *et al*. 31, which detected 1164 copies/mL of plasma. The specificity limit in terms of divergence from the tiled L segment sequence (531 nucleotides) was estimated at about 13–14%, a value exceeded by the undetected Iranian samples, and approached by the poorly detected China strain (13.7%), which lacked significant match by BLASTN analysis. The CCHFV isolate from Kosovo 429 and the Lassa virus strain AV, despite their low call rates (29.2% and 28.7%, respectively) were detected by BLASTN because they share, respectively, stretches of 21 and 25 consecutive nucleotides with the tiled sequence. This was not the case for the CCHFV isolates Iranian 407 (28.4%) and Turkey 09139 (33.5%) with similar call rates (28.4% and 33.5%, respectively) but sharing no stretches longer than 11 and 16 nucleotides. This indicates that the microarray may preferentially identify sequences that have stretches of ∼20 consecutive nucleotides identical to the tiled sequence, regardless of the overall similarity.

Apart from human samples, the potential of the microarray was also preliminarily tested in animal organ material. It was able to detect viruses in brain samples from experimentally infected animals. This has been demonstrated not only in mouse brain that was intentionally infected intracranially for virus isolation, but also in moribund *Saimiri sciureus* infected intravenously with the neurotropic Nipah virus. In both cases, the amplification of the viral sequences was not affected by the complexity of brain genetic material but a higher background was observed during the hybridization step (lower call rates).

Taking all results together, there is still room to enlarge the spectrum of pathogen detection by increasing the capacity of the microarray. This would allow not only the detection of all currently known isolates but also the discovery of new ones with reliable sequence information. To this purpose, the next-generation panvirological microarray, PathogenID v3.0, will include additional CCHFV sequences from the Middle East, Greece and Asian clusters, as well as geographical variants of families *Filoviridae* (Bundibugyo ebolavirus, Sudan ebolavirus and Ivory Coast strain), *Arenaviridae* (Ippy virus, Mopeia vrus, Mobala virus and Tacaribe virus), *Paramyxoviridae* (Tioman virus) and *Bunyaviridae* (Prospect Hill virus). This improved covering of the sequence space will allow detection of new emerging viruses substantially divergent from the tiled sequences.

## Supporting Information

**Table S1**: Raw sequences in FASTA format obtained following hybridization on the Pathogen IDv2.0 microarray of amplified viral RNA obtained from **(A)** cellular supernatants, **(B)** human sera and (**C**) animal brain. The sequences are listed following the same order of Tables 3, 4 and 5.
