# Supplementary material for: Molecular diagnostic and genetic characterization of highly pathogenic viruses: application during Crimean–Congo haemorrhagic fever virus outbreaks in Eastern Europe and the Middle East
Source: Clin Microbiol Infect. 2012 Dec 14;19(2):E118–28. doi: 10.1111/1469-0691.12075 (PMC3663000; doi:10.1111/1469-0691.12075)
Supplement: Supplementary file 1 [file clm0019-E118-SD1.doc]

**Table S1**:

Raw sequences in FASTA format obtained following hybridization on the Pathogen IDv2.0 microarray of amplified viral RNA obtained from **(A)** cellular supernatants, **(B)** human sera and (**C**) animal brain. The sequences are listed following the same order of Tables 3, 4 and 5.

**(A)**

>VirLassa:IP_mix4_4 Start=12 End=512

cctaaaagtgtcattggcaggtttgttgcagagtttaaatctagattttttgtttggggagatgaagtgccattattgacaaagttcgttgcagcagctcttcacaatataaagtgtaaagagccacaccaactggcagaaacaattgacacaatcattgatcagtcggttgcgaatggtgtccctgttcacttatgtaatctaatacagaaaagaacctnaaatcttttnnnntatgcaagataccctattgacccattcttattgaattgcgagacagatgttagagactgggtagatggcaaccgtagttataggattatgaggcaaatagagggattgattcctaatgcttgcagcaagattagatctatgttaagaaaactgtacaataggttgaagactgggcaactccatgaagagttcacaacaaactatttgtctagtgagcacctctcatcactaagaaatctttgtgagctgttggatgttgagcnnnca

>VirLassa:IP_mix5_5 Start=12 End=512

nnngnnnnnnngngnnncngnnnnnnnncngagtttaaangcgnnnntnnntnnnnnnnnnnnnnnnnnncnnnnntnnnnnnnnnnangncagcannnnnncnncnnnnnnnnnnnnnnnnnnnnnngnnnnnnncnnngncaattgacacaatcattgatcagtcagnggnannnnnagnnnntncnnnncccnnncnnnnnnnnnnnnnnnncnnnnnnnnnncnnnnnnnnnncncnnnnntnnnnntnnnnnnnntnnnnnnnnngnnnnnnnnnnnnngnnnnngnnnnngnncggcnnnnnnnntnncnnnnngcnnnngcnngnnnnnnccngnnngnnngcnnncnngnnncgnnngnnggnnnnnngnnnnnnnnnnngnnnnnnncnnnngnctgggcaacnccnnncganngccnannnccnncnnngtnncncncnncnnnnnncnncnnccnnnnnnnngngnnnnnnnnnnnnnnnnnnnnnnnnn

>VirLassa:IP_mix6_6 Start=12 End=512

cctaaaagtgtcattggcaggtttgttgcagagtttaaatctagattttttgtttggggagatgaagtgccattattgacaaagttcgttgcagcagctcttcacaatataaagtgtaaagagccacaccaactggcagaaacaattgacacaatcattgnncagtcggttgcgaatggtgtccctgttcacttatgtaatctaatacagaaaagaaccttaaatcttttgcaatatgcaagataccctattgacccattcttattgaattgcgagncagatgttagagactgggtagatggcaaccgtagttataggattatgaggcaaatagagggattgattcctaatgcttgcagcaagattagatctatgttaagaaaactgtacaataggttgaagactgggcaactccatgaagagttcacaacaaactatttgtctagtgagcacctctcatcactaagaaatctttgtgagctgttggatgttgagcnnnca

>VirJunin:IP_VHF_exp_30_Junin Start=12 End=515

ccaaagagtgtgataggaacttttgttgctgagtttaagncaagattttttgtaatgggtgaagagactcctttactcaccaagtttgtgnctgctgctctacacaatgttaagtgtaaaactccaacacaactgtcagagnctatagncacaatttgtgatcaatgtattgctaatggggttagcactcatatagtgtcaaagatttccattagagtcaaccaattgattagatactctggatacagagagactccgtttggagctattgaagaacaagatgtcaaagnctgggttgatgggtctagagggtaccgtctacagaggaaaattgaagctattttttctgatgataaggagncaatgtttataaggaactgcgcgnggaaagtgtttaatgacattaagagaggcaaaatctttgaggaaaaccttatcaacttgataagcaggngagnngatgaggctttatccggattcctgcaatatgccgggtgtagtgaa

>VirCrimeanCongo:IP_mix2_2 Start=12 End=518

aattcagtcagcttcttgttcaataaactctccagaaactcacctacagaagtaactgacatagttgttggagctataagtactcaaaaggttagaagttatctaaaggcaggaactgcaacaaaaacccctgtgtcgactaaagacgttctggagacttgggaaaagatgaaggagcacatactcaacagaccaacaggactgacactgcccaccagtttggancaggcaatgcgcaaaggactggtcgaaggtgtggtcatctccaaggaaggttctgagtcatgtatcaatatgttgaaggaaaatttggaccgaataactgncgaattcgagcgaacaaaatttaaacatgaacttactcagaatattaccacaagtgagaagatgctattgagttggttgagtgaagacatcaaatcatcgagatgtggtgagtgcctctcaaatataaagaaagccgttgatgaaactgccaatctatcagaaaagattgagctgctcgct

>VirCrimeanCongo:IP_mix4_4 Start=12 End=518

aattcagtcagcttnttgttcaataaactctccagaaactcacctacagaagtaactgacatagttgttggagctataagtactcaaaaggttagaagttatctaaaggcaggaactgcaacaaaaacccctgtatcgactaaagacgttctggagacttgggaaaagatgaaggagcacatactcaacaggccaacaggactgacactgcccaccagtttggaacaggcgntgcgcaaaggactggtcgaaggtgtggtcatctctaaggnaggttccgagtcatgtatcaatatgttgaaggaaaatttggaccgaataactgacgaattcgagcgaacaaancttaaacatgaacttactcagaatattaccacaagtgagaagatgcttttgagttggttgagtgaagncatcaaatcatcgagatgtggtgagtgcctctcaaatataaagaaaaccgttgatgaaactgccaatctatcaganaannnngagctactcgct

>VirCrimeanCongo:IP_mix1_1 Start=12 End=518

annnnnnnnngnccnngnnnnnnncnnnnnnctnngnnctcacctacagaagtaactgacatagttgttggagctataagtactcaaaaggtnnngngnnncnngnnggcagggactgcaacaaaaannnctgtgtcgacaaaagacgttctggagacttgggaaaagatgnnnnagcannnnntcaatnngnnnnnnnnntnncnnnngnnngncagtttggaacaggctatgcgcaaaggactggttgaaggtgtggtcatctctaaggnangnnnngnanncnnnnggnnnnngngnnnagnaaatttggaccgaataactgatgaattcgagcgaacaaaattccngnnnnnnnncgcncannnngnnggnnnngnnnnnnnnnngncnnngnncnggntgagtgaagatatcaaatcatcgagangnncnnnnngnnngnncnnnnnnnnncnnacnnnggncgnnnntgnnncnnncnntnnngnanngnnnnnngngngc

>VirCrimeanCongo:IP_mix3_3 Start=12 End=518

nnnnnnnnnnnncnncgnnnncnncnnnnnnncnnnnnnnnngnnncnccnnctnccnnnnggnggnnngnnnncgnnnnnnncnnnnnnnncgnnnnnnnnnnnnagncnnnnnnnnnnncnaaaancnctgtgnngncaaaagncntnntagnnnnnngnncnnnnnnnnnncnncnnnnnnnncnnnnnnnnnnnnnnnnncnnnngnnnncnnnnnnnncgnnggcagngnnnagnnnnctggnnnnannnnnggncanctccaagncgnnngnnnnannnnnnggncnnnnnngncngnnnnnnnngnnnnnnnnnnnnncnnncnnncnnncgncaaaatnnnnncnnncgnncgcnnnncnntcngnnnncgcncnnnnnnngncgnngnnnnnncganntgngnnnngnnnnnnnggnnnnnnnnnnnncngnnngnnnnnnnnncnncnngcnnnggnngnnnctgcnaccnnnnnngncnnnnngnncnnnnngngc

>VirHantaan:IP_mix7 Start=12 End=497

cacatcaaacagtttttagattttttccctgatgggcaccangnnnaagngaagggaaactggctgcngggtaacttgancaagtgntcttcacttttcggtgttgcaatgtcattactatttaaacaggtatggactaacttattccctgagcttgattgtttctttgagtttgcacatcacnctgatgatgcattattcatttatggatacttggaaccagtagatgatgggactgnctggtttttgtttgnctcacaacagattcaagcaggccatttgcactggtttagtgtaaatacagagatgtggaagagtatgtttaatctacatgagcatatacttcttctaggctccatcaagatctcaccaaagaaaactacagtgtcncngacaaatgctgaatttttatcaacattttttgaaggttgtgctgtatcaataccatttgttaaaatactcttaggttctctatcagacttacca

>VirSinNombre:IP_HFV1 Start=12 End=515

gtttcactatatagcacaaaggaaaatattgggttacaagatgatgttgcagctacacgattgtgtatagaagtctggagatggtgcaaagctaatgatcagaatgtaaatgactggttaaatgcactgtactttgaaaagcaaactctcatggattgggtagaaaggttccgtagaaaaggagtggttcctattgaccctgaaattcagtgtattgccttactactttatgatgtgttaggttataagagtgtattgcagatgcagncaaataggcgagcatattcagggaaacagtatgatgcatattgtgttcaaacgtataatgaagaaacaagactctatgagggagatttaagggtcacatttaattttggccttgattgtgcaaggttggaaatcttttgggataagaaagaatatattcttgaaacatcaattacgcaacgtcatgttttaaagttgatgatggaggaagtcacacaagaattgttaaggtgtggg

>VirSeoul:IP_mix6_6 Start=12 End=539

catgtatataagnctnntttttatatgtctagaaaacttagacactatattgattctatggncacttatgaacctcatgtcagagactttttgaatttctttccagacgggcatcatggagaggtacgaggcaattggttgcaaggtaacttgaacaagtgttcatcattgtttggtgtggcaatgtccttgttatttaaagaaatttggacaaggttatttccagaattggactgcttttttgaatttgctcatcattcagatgatgcacttttcatatatggctacttagaaccagctgatgacgggactgattggttcttatttgtatcccagnannnacaagctggaaaattgcattggtttaatgtaaatacagagatgtggaaaagtatgtttaatctgcatgaacacattcttctgttagggtcaattaaaatatccccaaagaagacaacattatcgccgacaaatgctgagtttttgnctacattttttgagggttgtgctgtatcaattccctttatt

>VirDobravaBelgra:IP_mix9_2 Start=12 End=518

cgtnntnncanacataaaangnannngnannnnnnatntattcttggtnnnncnnnnnntcngnnnnncttatcaaaagatacattccagcatgaaagnttgggtnnnnncnnattcatagggaaagtgcaatggaaaatattnatnnnnnggnnnnnantnnngttttttgncatgnacacnnntnngnnnnnnnnnnngngnngtgnncaacatgtaacnnnngattacattattccaaagggnngagncnnnnnnncnnntnnnnnngttaggaaaataaatgatccaagcatagtcactgctntgacaatgcaatcaccncnccagttaagattcagaangcaagcaaagcnncacatgaaagttngcaaattggatgatgncngnnnnncatttagggagatcttagcngntnnnnncngnnnnccngntncncngnnnnnngnngngncnnnntcgnncnntttncagacactnncaancnnngnngncngnnnnnnnnnn

>VirRiftValley:IP_mix6_6 Start=12 End=536

ataaaggaatcagatggtaagacaaaaaccaacttgattggaatccttcagagatttctagatggtgatcacgttgtcnncataattgaagagatgggagccggaacagtgngtggattcatcaagagacaacaatctaaagttgtgcagaacaaagtggtctattatgnanntgggatttggagaggcttcatggatggatatcaggtccatctagagatagaaaatgacataggacagcnnncaaggcttaggaatgtcacaactaactgtcagagcagcccatgggacctgagtattccaataaggcaatgggcagaagncatgnnggtcacaaacaaccaggattattcctctaaatctagcaggggngccagatattggatgcattcattcaggatgcaaggacctagcaagccatttggatgcccagtttatattattaagggtgatatgtcagatgtcatcagactgagaaaggaggaggtggagatgaaagtacgnggctctactctcaacttgtac

>VirKyasanurForest:IP_mix1 Start=12 End=491

gcannncgngnnnnnagggaagctgatgtggccgaaagaataacnnnnnngnnancnnnnngngnngnnnnnnnncntnngnntaaagaacatccatacagaacatggcaanncnnnggnngtnnnannncannagccncaggatnngctgcntctttgatcaatggnnngncnnagcttctnanntggccttggaatgctaggnaggatgtggtgcgcatggcaatganacntnncgnnnnanntngncaacannnngntntcnaggnnnaggtggataccaaagcgcaggagccacaggtgggaaccaagatcattatgaggncnnnccntnnnngncnnnncggnnnnntcgcgggaaagaaaacaccacgacngngcacgcgagaanaattcatcgcaaaggtgcgntcaaacgntgcgctcggagnttggtcagatgaacaaaatcgctgntancnnntnnnngnagcagtggag

>VirTickBorne:IP_mix3_3 Start=12 End=488

nnngncaaggtganagancncnnnnngncnnnnngnnnnnnnnnnnnnnancnnnnnnncagtnntnnnnggannnngnntnnnncnnnganannnnnanngnggcngnncnggggtnnnnnnnnnnnnncncaannnnncnnngnngnnnnnnannntnnngnnccnnnnnnnnncnngtnnnggncgtggaatgcacgggaagatgtggtgcngntggcannngnnnnnnnccnngcnttcggncngcagagagtgtncnnggnccnannngacaccnnggcacagnagccnnngcnannncncnngnccannnnnnnnnnngngnnngnnnnncnnnnnnnnnngnngnnnnnnnnnnnnnnnnnnnntnnnnnnngcagagaannnnnnnntgcnnnnnnnnnnnnnnnnnnngncnnnnnnnncnggtctgatgagcaaaacnaatgggcnnnngnnnnnnngnnagngnnn

>VirRestonEbola:IP_mix2_2 Start=12 End=515

gaatatcctcctgaaggcccgagttcgtaccgagggcacttaggaggcatagagggattacaacaaaaactgtggacgagtatatcctgtgcacaaatctccttagtggaaattaaaactggttttaagttacgatcagcggtcatggnngacaatcagtgtataaccgtattgtctgtttttccacttgaaacngaccctgaagagcaggngcaaagcgccgaagacaatgctgcaagagtagcagcaagtctngcaaaagtaaccagtgcatgtgggatctttcttaaaccagatgagacatttgtacactcaggtttcatttatttcggaaaaaaacaatatctcaatggtgtacaattaccgcaatcactcaaaacagcagcaagaatggcaccactctctgatgctatattcgatgatctacaaggaacacttgccagtattggaactgccttcgaacgtgctanancggaaacgcgacatatcctcccatgtcgtatt

>VirZaireEbola:IP_mix7 Start=12 End=515

gacaacnnnncnnangggcccagttcanacagaggtcatatgggagngattgaaggactgcaacaaaaactctggacaagtatttcatgtgctcaaatttctttagnngaaataaagactggttttaagttacgctcagctgtgatgggtgacaatcagtgcattnccgttttatcagtcttccccttagagactgacgcagacgagcnggaacagagcgccgangncaangcagcgannntgnccgncngccnagcaaaagttncaagtgcctgtggaatctttttaaaacctgatgaaacatttgtacattcaggttttatctattttggaaaaaaacaatatttgaatggggtccaattgcctcagtcccttaaaacggctncaagaatggcaccattgtctgatgcaatttttgatgatcttcaagggaccctggctagtatnggcactgcttttgaacgatccatctctgagacacgacatatctttccttgcaggata

>VirZaireEbola:IP_mix8 Start=12 End=515

gacancnnnnnnnnagggcctagttcatacaggggtcanntgggagggnttgaaggactgcaacaaaaactctggacaagtatttcatgtgctcaaatttctttagttgaaattaagnctggttttaagttacgctcagctgtgntggncgacaatcagtgcattnccgttttntcagtcttccncttagagactgacgcagacgagcaggancagagtgccgaagacaatgcagcganngtggccgntngcctagcaaaagttncaagtgcctgtggaatctttttaaaacctgatgaaacatttgtncattcaggttttatctattttggaaaaaaacaatatttgaatggngtccaattgcctcagtcccttaaaacggctacaagaatggcaccattgtctgatgcaatttttgatgatcttcaagggacccnagctnntnnnggcaccgnnnnnnnncngtccantnntgagacacgacatatctttccttgcaggata

>VirMarburg:IP_mix4_4 Start=12 End=515

aataacccaccggattgtgctaatgcttatcattatcacttagggggtatagagggacttcaacagaaattgtggacatgtatatcatgtgcccagatcacccttgtagagttaaaaactaaattaaaattaaaatccagtgttatgggtgataatcaatgtataacaactctaagtctttttccaattgatgctcccgacgattatcaagagaacgaagctgaattaaatgcggcacgagtngcngtcgaattagctattactacgggnnnnngtggtatatttttgaagcctgaagaaacatttgtccattcagggttcatttattttggtaaaaagcaatacctcaacggtgttcaactgccacaatcattgaaaacaatggcaagatgtggacccttatctgactctatttttgatgatcttcaaggttcccnggccagtattggtacatcctttgagagaggaacaagtgagacacggcacatttttccgagtcgttgg

>VirMarburg:IP_mix5_5 Start=12 End=515

aataacnnaccggattgtgctaatgcttatcattatcacttagggggtatagagggacttcaacagaaattgtggacatgtntatcatgtgcccagatcacccttgtagagttaaaaactaaattaaaattaaaatccagtgttatgggtgataatcaatgtataacaactctaagtctttttccaattgatgctcccgacgattatcaagagaacgaagctgaattaaatgcggcacgagttgcngtcgaattagctattactacgggngnnggtggnatatttttgaagcctgaagaaacatttgtccattcagggttcatttattttggtaaaaagcaatacctcaacggtgttcaactgccacaatcattgaaaacaatggcaagatgtggacccttatctgactctatttttgatgatcttcaaggttcccnggccagtattggtacatcctttgagagaggaacaagtgagacacggcacatttttccgagtcgttgg

>VirNipah:IP_09_04_29_mix2_2 Start=12 End=515

gaaaaaactcctgaagatgatatattcattcattatcctaaaggcggtattgaaggatatagccaaaaaacatggactatagcaactatcccctttttattcttgagtgcctatgagacaaacacgaggattgctgcaattgtccaaggagacaatgaatcaattgctatcactcaaaaagttcatcctaatcttccctacaaggtaaagaaagagatctgtgcaaagcaagctcagctttattttgaaaggttaaggatgaacttaagagncctcggccacaatcttaaagctacagaaactatcatcagtacacatctttttatttattcgaagaaaattcattatgatggtgctgtgctgtctcnggcactcaaatcaatgtcaagatgttgcttttggtcagagactctggtggatgaaactagatcagcttgtagtaacatcagcactacaatagctaaagctatagaaaatgggttgtcaagaaatgtcggctattgc

>VirHendra:IP_mix1_1 Start=12 End=515

gatgatactcctgaagatgacatatttatccattcaccaaaaggcgggattgaaggttatagtcagaagacttggacgatcgcaacaattccctttctgtttctcagtgcctacgaaacaaatacaaggattgcagctatagttcagggagncaatgagtcaattgccataactcagaaggtccaccctaatttaccttacaaagtcaagaaagaaatttgtgcaaggcaagctcaattatattttgacaggctcagaatgaatcttagggccttaggacacaacttaaaagcaacagagaccattataagtacacacttatttgtctactctaagaaaatacattatgatggtgcagtattatcacaagccctcaaatcaatgtctaggtgttgtttttggtcagagncattggtggacgagncaagatctgcttgcagcaatattagtactaccatagctaaggcgattgagaatggattatcaagaaatgtgggttactgc

**(B)**

>VirCrimeanCongo:IP_mix2_2 Start=12 End=518

aattcagtcagcttcttgttcaataaactctccagaaactcacctacagaagtaactgacatagttgttggagctataagtactcaaaaggttagaagttatctaaaggcaggaactgcaacaaaaacccctgtgtcgactaaagacgttctggagacttgggaaaagatgaaggagcacatactcaacagaccaacaggactgacactgcccaccagtttggancaggcaatgcgcaaaggactggtcgaaggtgtggtcatctccaaggaaggttctgagtcatgtatcaatatgttgaaggaaaatttggaccgaataactgncgaattcgagcgaacaaaatttaaacatgaacttactcagaatattaccacaagtgagaagatgctattgagttggttgagtgaagacatcaaatcatcgagatgtggtgagtgcctctcaaatataaagaaagccgttgatgaaactgccaatctatcagaaaagattgagctgctcgct

>VirCrimeanCongo:IP_mix4_4 Start=12 End=518

aattcagtcagcttnttgttcaataaactctccagaaactcacctacagaagtaactgacatagttgttggagctataagtactcaaaaggttagaagttatctaaaggcaggaactgcaacaaaaacccctgtatcgactaaagacgttctggagacttgggaaaagatgaaggagcacatactcaacaggccaacaggactgacactgcccaccagtttggaacaggcgntgcgcaaaggactggtcgaaggtgtggtcatctctaaggnaggttccgagtcatgtatcaatatgttgaaggaaaatttggaccgaataactgacgaattcgagcgaacaaancttaaacatgaacttactcagaatattaccacaagtgagaagatgcttttgagttggttgagtgaagncatcaaatcatcgagatgtggtgagtgcctctcaaatataaagaaaaccgttgatgaaactgccaatctatcaganaannnngagctactcgct

>VirCrimeanCongo:IP_mix1_1 Start=12 End=518

annnnnnnnngnccnngnnnnnnncnnnnnnctnngnnctcacctacagaagtaactgacatagttgttggagctataagtactcaaaaggtnnngngnnncnngnnggcagggactgcaacaaaaannnctgtgtcgacaaaagacgttctggagacttgggaaaagatgnnnnagcannnnntcaatnngnnnnnnnnntnncnnnngnnngncagtttggaacaggctatgcgcaaaggactggttgaaggtgtggtcatctctaaggnangnnnngnanncnnnnggnnnnngngnnnagnaaatttggaccgaataactgatgaattcgagcgaacaaaattccngnnnnnnnncgcncannnngnnggnnnngnnnnnnnnnngncnnngnncnggntgagtgaagatatcaaatcatcgagangnncnnnnngnnngnncnnnnnnnnncnnacnnnggncgnnnntgnnncnnncnntnnngnanngnnnnnngngngc

>VirCrimeanCongo:IP_mix3_3 Start=12 End=518

nnnnnnnnnnnncnncgnnnncnncnnnnnnncnnnnnnnnngnnncnccnnctnccnnnnggnggnnngnnnncgnnnnnnncnnnnnnnncgnnnnnnnnnnnnagncnnnnnnnnnnncnaaaancnctgtgnngncaaaagncntnntagnnnnnngnncnnnnnnnnnncnncnnnnnnnncnnnnnnnnnnnnnnnnncnnnngnnnncnnnnnnnncgnnggcagngnnnagnnnnctggnnnnannnnnggncanctccaagncgnnngnnnnannnnnnggncnnnnnngncngnnnnnnnngnnnnnnnnnnnnncnnncnnncnnncgncaaaatnnnnncnnncgnncgcnnnncnntcngnnnncgcncnnnnnnngncgnngnnnnnncganntgngnnnngnnnnnnnggnnnnnnnnnnnncngnnngnnnnnnnnncnncnngcnnnggnngnnnctgcnaccnnnnnngncnnnnngnncnnnnngngc

>VirCrimeanCongo:IP_VHF_exp_9_CCHF_090137 Start=12 End=518

nnngnnnncncncnnnncnnnnnnnnnnnngntnnnnnnncacctacagaagtaactgacatagntgttggagctataagtactcaaaangnnnnnnnnnncnnnnaggcaggaacngcaacaaaaannncngtnncnnnannngntnnnctggagnctnggnnnnnannnncnnnnnnntnnnnnnnnncngnnnnnnnnnnccnnnntnnngncagtttggancaggcaangngcaaagnnnnnnnnnnnnnnnnnnnnanctntaaggnnntngnnnnannnnnnnnnnnnnnnngnnnanaaaatttggaccgaataactgatnnnnncgagcgaacaaaanncnancnngaacttactcaganngangnnnnnnnannanncnngnnggnnnncnnnnnnnnngnagntancaaatcancnnnnngnnnnnnccnnnggnnnnnnnanannnntnnnnnngnnnnnnnngnnncanannnngnnnnngngnnnnnnnctnnn

>VirCrimeanCongo:IP_VHF_exp_11_CCHF_090139 Start=12 End=518

nnnnnnncnnnnccnngnnnncnncnnnnnnnnnnnnnnnnannnncagangnnactgncagngttgttggagcnannagnnctcaaaangnnnnnnnnntnnngnnncntnnnnnnnnanaanaannnnnnnnnngnnnanangncntgctggagnnnngncnnnannnnnnacnncnnnnnnnncnnnnnnangnnnnncnncnnctgnccacnnccnnngcnnagncnngnnnnnnnncnnnggnnnnnnnnnnnnnnngnngnnnnncantcgnnnnggncnncggnnnnnngnnnnnnnnnnnnnngnanngnnnnnnnnntnnnnnnnagcnaannnncnnnnnnnngnnnnnnnnnnnccnnnnngnngnnnnngannncnngncgnnnnnctntnngagngnagntatcaaatcannnannngnncnnncnnnnngngncnannncnnnnnnnnnnggnnnnnnctnncnnanncnnngncgnntnnntnnnngnnngc

>VirCrimeanCongo:IP_FHV_Kosovo_422_9 Start=12 End=518

nnnnngnnnnnnnnnnnngnncnnnnnnnnnccnggnnnncnnncncngaagtaacngacatngttgntggagctatnagtnctcaaaannncnnnnnnnnnnnnnnngcnggnnngcnnnnnnaanccctnnnnnnnnnannnnacgttctggagncnnngnnnnnatnnnnnnnnnnnnnnnnnnnnnnnnnnnnnnnnnnncnnnnncncncnnnnnnnnnncnggnnnngngnnnnnnnnnanntnnnggngnggtcatcnctnnggnnnnnnnnnnannnnnnnnnnnnnnnnnnnnanaaaatttggaccgaataactgatgaattcgagcgnnnnaaatttaaacatgaacttactcagaatgnnnnnnnnnnnnnannngnnnnnnntnncnnncggngtgaagatatcaaatcannnnnnnnnncnnnnngnnngnnnnnnnannnnnnnnnnngnnnnnnnnnnnnnnnnnnnnnnnnnnnngngnnnncncnntnn

>VirCrimeanCongo:IP_FHV_Kosovo_423_10 Start=12 End=518

nnngnnnnnngcnnncgnnnnnnannnannnnntnnncngnnnnnnnnnnnnnnnntgcnnnnnnnnntggngnnnnnngnnctcaannnncgnnnnnnnnnnnnnnnnnnnnnnnnnnannnaaannnnnnnnnnnnnnnnnnnnnnngntggnngnnnnncnnnncnnnnnncnncnannnnnnnncnnnnnnnnnnngnnncnnnngcncncnnnnntnnnnnannnngnnnatnnnncnnngnnnannnnnnnnnnnngnngncnnnnnnnngnncnnnnnnnnngnnnnnnnnnncnggnanncnngnnncnnannncngntnnnnnnnnncgaacaaaantnnannatgnncncgnnnnnnnngtngnannnnnnnnnnncnnnnngnnnnnnnntcnnnnngcnnnnnnnnnnnnnnnnnannnngnnnnnngnnnnnnnnnannnnnnnnnngntnggnnnnnnnntncnnannnnnnncnnnancgntnnnnnngnnc

>VirCrimeanCongo:IP_FHV_Kosovo_427_11 Start=12 End=518

anntcagnnngnnnncgcnnnnnnnnnnnctcanggaactcacctacagaagtaactgacatagttgttggagctataagtnctcaaaaggttaggnnnnncnnnnaggcagggactgcaacaaaaannnctgtgtcgacaaaagacgtnctagagacttgggaaaagatgaaagagcacannctcaatangnnnnnnncntnacnncnnnnnnnnnnnnnggngcaggctatgcgcaaaggactggttgaaggtgtagtcatctccaaggaaggttctgagtcatgcnnnnnnnnnnngaaanaaaatttggaccgaataactgatgaattcgagcgaacaaaattccngcangaacttactcagaatgnnnnnnnnnnntnacgcnnnnngatgagctggttgagtgaagatatcaaatcatcgagntgnncnncnnnnnngnncnnnnnnnnnnnaaccgttgatgagnnnnngncnnnnnnnnnnnnngnnnnnncagnnnnn

>VirCrimeanCongo:IP_FHV_Kosovo_429 Start=12 End=518

nnnnnnnnnnnncnnngnngnnnncnnnnnnccnngnnnnnnnncncagaagnaactgncatagttgttggagctataagnnctcaaaangncgannnnnncnngnngncnnnnnnnnnannnaaannnncngtnnnnnnanaagacgtgctggagncnngnnnnnnnnnnnnncnnnnnnnnnnncnnnnnnnnnnnnnnnnncnnnngnnnnnnnnnnnnncnnagnnnnnnnagnnnnnnnnnnnnnnnnnnnnnnnnngnngannnccnntngnnnnnnncnnnngnnnnnnnngnctnnnannnntgnnnngnannnnngnnnnnnnnnnncnaacaaannnncnnnnnnnncnngcnnnncnnnnngnnnnngnnnnnnnnnngncnnngnnnnntnnnnnngcgnnnnnnnnancngnnnnnnnngnnnnnngnnngngcnnnnnnnnnnnngnnnnggnngnnnnngnnncannnnnngcnnnnnngnnnnnngngnnc

>VirCrimeanCongo:IP_FHV_Iran_397_2 Start=12 End=518

nnnnntncgnnnccnngnnnnnnnncnnnnggntannnnnnnnnnnnnnnnnnnnnngnanngnnnnnnnnngncgncnncnnnnnaaaggtnngnagttntcnnnnnnnnannnnnnnanaanaannnnnnnnnnnnnnnngnnnnncgnngnnnnnannnnnnnnnnnnnnnnnnnnnnnnnnnnnnnantnntnaannnncnnnaagnnnnnnnnnntnnnnnagnnannnnnnaaannnnnnnngnnnncnnngnnnnnnngannnccnntngnnnnnngnnnnnnnnnnnnnnnncagnaaannnngnncngaannannnntnnnnnnnnnnnnnnnnannnncnnnnnncnnncncnntcnnnnnngnnnnnnnnnnnnnnnngncnnnntnntntnnnnnngnnncnnnnnnnnnnggngncnnnnnnnnnngnnngngnnnannnnnnnnnnnnnaggnngnnnntgcnanannnnnnnncnnannnnnnnnnnngcnc

>VirCrimeanCongo:IP_FHV_Iran_402_5 Start=12 End=518

nnnnnnnnnnnncctngnnnnnnncnnnnnnnnnnnnntngngnnnnnnnnacgnnnnnnnnnngnntnnnggnnnnnnncactcaaaaggttagannnannnnnnngnnnnnnnnnnnanannaannnnnnnncnnnnnnnnnnnnnngnnggnngnnnnnnnnnnnnnnnnncnnnnnnnnnnnnnnnnnnnnnnnnnnnnnnnnnnnnnntnnnnntnnnngnagncaatgngcaaagnnnnannnnnnnnnntggtcanctnnaanncnntcgnnnngnnnnnnngnnnnnnnnnnnnannaaatntggaccgaataacngntnnnnnnnnnnannnnnnnnnncnnnnnncgnncnnngnnnnntnnnnngnnnnngancnnnngnnnnngnnntntnnnnnngcnnnnngnnnannnngnnnnnnnnnnnnnnggnngnnnnnngnnnnnnnnnnnnnggnngaanctgnnnnannnnnnncnnnatnnnnnnnnnnnnnc

>VirCrimeanCongo:IP_FHV_Iran_406_6 Start=12 End=518

nnngnccgnnnnnnnngnnnnngcnnnnnnggnnnnnntnnngnnnanncnnnnngnnnnnngnnnnnnnnnnnnnnnnncncncaaaaggttagnnnnnnnnnnnngccnnnnnnannnnnnnnnnntnnnnnnnnnnnncnnnnnnnnnnnnnnnnnnnnncncnnngnnnncnnnnnnnnnnnnnnnnnnnnnnnnnnnnnccncngnnnnnnnnnnntgnnaagnnanngngnaaagnnnnannnncnnnnnngntcannnnnannccnntnnnncngnnnnnnnnnnnannncnnnnannnngtntggaccgaataacnnntnnnnnncnnnnnnnngannnncnnnnnnngnnnnnnnnnnnnnngnnnnnngnnnnnnncnngncnnnnngnnntcnnnanncnnntnnnnnnnnnngcnannnnncncnnngnnngnnncnnnnncnnnnnnnnnnngnngannntgnnncagannnnnnngnnnngnnngnnnnnnnn

>VirCrimeanCongo:IP_FHV_Iran_407_7 Start=12 End=518

nnnnnncnnnnnnntnnnnnnnnannnnnnnnnnnnnntgnnncnnnnnnnnnngnnntnnnntnnttnggggcnnnnnncnctcaaaaggttagnnnnntnnncnnncnnnncnannnnncnnaanannnnnnnnnnnnnnnnnnncngnnngnngnnnnnnnnnnnnnnnnncnnnnnnannncnncnnnnnnnnannnnnnnnnnnnnntnnnannttngcnnnnncaannngnaaaggnnnannnnnnnntgtggtcatctnnannncnntngnnnngnnngnnnnncnnnnncnnnnanaaaatttggaccgaatnactnntnnnnnnannncnnnnnnannnnnnnnnnngnnnnnnnnnnnntngnnngnnnnnnnnnnnnngnngnngngtnnnnnnnnnncgnnncnnnnnanngncnannnngnnnnnngnnngnnnnnnnnncncnnnnnaanggnggannnngncnnannnnnnnnnnnanngnnatnnncnnnc

>VirCrimeanCongo:IP_FHV_Iran_409_8 Start=12 End=518

nnnnnnnnnncnccnnnnnggnnacnnnnnnncnngnnnnnnnnannnncnnnnnnngnntnnnnnnannnngnnnnnnncnnncanaaggnnngnnnnnnnnngnnnnanannanannannnnnannnnannnnnnnnnnnnnngnnngnnngnngncgnncnnnnnnnnnnncncnnnnannnnnnnnnnnannnnncnnnccnnnnnnnnnnnnnnnttnnnnannnannnnngannnnnnnnnngannnnngngcncannnnnannnnnntnnnnnngnnnnnnnnnnnnnnnnnnnnnnnnnnngtgnnncganannnnnntnnnnntcnnnnnnnnnannnncnnnnnncnnncnnnnnnnnntnngnannnagtgannnnnnnncnnnnnnnnntcnnnnnccnnnnnnnnnnnnngnnnannnngnnnnnnggnngngnnnngnncnnnnnnnnnnggnngnnngtgnnncannnnnannnnnannnnnnnnnnnnngc

**(C)**

>VirCrimeanCongo:IP_HFV7 Start=12 End=518

aattcagncancttcttgttcaataaactctccagaaactcacctacagaagtaactgacatagttgttggagctataagtactcaaaaggttagaagttatctaaaggcnggaacnnnaacaaaaannnntgnancnnctaaagacgttctggagacntgggaaaaantgnaggagcacannctcaacaggncancagnnnngccncngnnnnccagnttggancaggcgnngnnnanangncnggncgaagntgnggtcancnctaagccngnnnccgagncatgtatcaatatgttgaaggaaaatttggaccgaataactgacgantncgagcnaacaaancttnancatgaacttactcagaatattaccacaagtganaagntgcntttgagttggttgagtgaagacntcaaatcatcgagnngtgnngngtgcctctcaaatatnaagaaaacngttgntgaaactgccaancnannnnangnntngnnnnnannngct

>VirNipah:IP_VHF_exp_12_Nipah_brain Start=12 End=515

gnaaaaactccngaanntnnnnnnnncnnncattatcctaaaggcggtattgaaggananagccaaaaaacatggnctanngcnnctatcnnctttttattcttgagtgcctntgagacaaacacgaggnnngctnnnnntgnncaaggngncaangaatcaattgctatcactcanaaagntcanccnnatcttccctacaaggnnaagaannannnnngngnnannncnnnncannnntnttttgnaaggttnaggangnannnnnnnnnnnncnnccacaatnnnaanncnncagaancnatcnncagnncacannnttnnntnnnnnnnnngnaaattcannnnnnngnngnnnngnnnnnncngncncncnnnncantgncnngntnnngctnntggnnngnannnnnggnggntgnaacnngnncagcnngnngnnacancagcacnncaannncnnnnnnnnnngaaaatgggnngncaagaaatgncggcnnntnc
